# Synthesis of Acidic Phosphonic Chitosan and the Complexation of La(III) in Acidic Aqueous Solution

**DOI:** 10.3390/polym17101341

**Published:** 2025-05-14

**Authors:** Min Zhou, Zhenglin Liu, Dandan Lu, Jiajun Wang, Zili Chen, Yunren Qiu

**Affiliations:** School of Chemistry and Chemical Engineering, Central South University, Changsha 410083, China; 222312163@csu.edu.cn (M.Z.); 232312186@csu.edu.cn (Z.L.); ludandan0707@163.com (D.L.); 242312177@csu.edu.cn (J.W.); zlchen0212@outlook.com (Z.C.)

**Keywords:** rare earth separation, phosphorylated chitosan, complexation–ultrafiltration, acidic aqueous solution

## Abstract

Due to the similar physicochemical properties of rare earth ions, their separation presents significant challenges. In this study, acidic phosphonic chitosan (aPCS) was prepared by modifying chitosan with phosphite and formaldehyde for improving the water solubility and complexing ability of rare earth ions in acidic aqueous solutions. DFT calculations revealed that its phosphonic groups serve as preferred reaction sites, forming stable bidentate complexes with rare earth cations. The complexation abilities of aPCS and phosphorylated chitosan (PCS) for La(III) were compared at various pHs by complexation–ultrafiltration. The results showed that aPCS achieved a 97% rejection for La(III), while 70% for PCS at pH 5 and P/RE 10. Furthermore, aPCS maintained higher rejection than PCS at pH of 3 to 7. In conclusion, aPCS demonstrates excellent potential for the selective extraction and purification of rare earth ions.

## 1. Introduction

As a national strategic resource, rare earth elements have become crucial in many areas of contemporary society [1]. However, the physical and chemical properties of rare earth elements are strikingly similar, especially considering that their ionic radii change minimally with increasing atomic number [2,3,4]. This subtle variation makes the separation of these elements a formidable challenge. At present, different techniques have been innovated for separating rare earth elements including ion exchange, solvent extraction, membrane, ion imprinting [5]. Among these technologies, complexation–ultrafiltration (C–UF) is an effective way to separate rare earth elements. The process involves complexing rare earth ions with water-soluble polymers that have nitrogen, sulfur, or carbonyl functional groups [6]. These polymers form large molecular coordination compounds with the rare earth ions. An ultrafiltration membrane with a molecular weight cut-off higher than that of the coordination compounds is then used to retain these large complexes, while allowing other free ions to pass through. This method effectively reduces the concentration of rare earth ions or selectively separates them from the solution [7,8].

Complexing agents are integral to complexation ultrafiltration. The challenges posed by the similar chemical properties of rare earth elements and the limited selectivity of current agents have driven extensive research into the development of more efficient complexing agents. It has been observed that rare earth ions primarily form ionic bond complexes with most inorganic ligands. However, complexes formed with phosphorus ligands exhibit a covalent bond structure that resembles a chelate form, rendering them more stable than complexes with other inorganic ligands [9]. Amide extractants are particularly promising due to their ease of synthesis, resistance to hydrolysis, complete combustibility, and minimal environmental impact. When compared to amines and phosphorus extractants, amides possess an N-C=O functional group that allows for electron delocalization across the nitrogen, carbon, and oxygen atoms. This delocalization increases the electronegativity of the coordinating atoms, thereby enhancing their interaction with rare earth elements and improving the stability of the resulting complexes. Additionally, previous studies have demonstrated that acyl and amino-α-phosphamide compounds exhibit superior performance with rare earth ions through biocomplexation, showcasing both excellent selectivity and coordination capabilities for rare earth elements [10].

Chitosan is the second-most abundant natural organic polymer, after cellulose. Due to its non-toxic and harmless biological characteristics, chitosan has promising applications in high-tech fields such as medicine, cosmetics, environmental protection, and biomedical materials [11,12]. However, the adsorption capacity of chitosan for heavy metals is relatively low, and its molecular rigidity and solubility are poor, which greatly restricts its range of applications. Chitosan contains a large number of amino and hydroxyl groups, but due to steric hindrance and differences in charge, most amino groups are in a free state, exhibiting higher reactivity [13]. Consequently, despite the greater number of hydroxyl groups, modifications to chitosan typically occur at the amino sites.

By modifying chitosan, its physicochemical properties can be improved, resulting in modified chitosan materials with superior mechanical strength, good stability, high selectivity, and enhanced adsorption capacity [14,15]. This significantly broadens the application fields of chitosan and its derivatives. Additionally, modification of the amino groups in chitosan tends to improve its water solubility more effectively than modification at the hydroxyl sites [16]. For example, Tan et al. [17] modified hydroxypropyl chitosan to obtain N-acylated chitosan derivatives, demonstrating that the reactivity of amino groups in chitosan is higher and more easily acylated compared to hydroxyl groups. Zhang et al. [13] successfully synthesized an amino-modified tailings/chitosan composite, which was then applied as an adsorbent for the effective removal of Pb(II) and Cd(II) from aqueous solutions.

Although phosphonic chitosan derivatives have been previously studied for rare earth ion removal, most of these works primarily focus on their use as adsorbents [18,19]. Such adsorption processes are inherently restricted by the available contact area between the solid adsorbent and metal ions in solutions, which often results in lower efficiency. Reduced complexation efficiency or insufficient selectivity under acidic aqueous solution environments is another challenge, which is more relevant to practical extraction and separation processes. Addressing these challenges is crucial for advancing rare earth recovery technologies.

In our previous work, we synthesized phosphorylated chitosan (PCS), which exhibited superior rare earth ion complexation performance compared to polyacrylic acid sodium (PAAS) [20]. However, its complexation efficiency was limited in acidic aqueous solutions, a common environment in rare earth separation processes. To address this limitation, we developed an acidic phosphonic chitosan (aPCS) by modifying the amino groups of chitosan with phosphating agents. This modification not only enhanced the water solubility of chitosan but also significantly improved its complexation efficiency under acidic conditions. Characterization of aPCS using Fourier-transform infrared spectroscopy (FTIR), nuclear magnetic resonance spectra (NMR), X-ray diffraction (XRD), and scanning electron microscopy (SEM) confirmed its structural properties. C–UF experimental results demonstrated that aPCS achieved superior rejection rates for rare earth ions, surpassing the performance of PCS under challenging acidic conditions. This study aims to address the challenges of rare earth separation in acidic aqueous solutions by exploring the potential of aPCS as an efficient complexing agent.

## 2. Materials and Methods

### 2.1. Materials and Membrane

Chitosan with an average molecular weight of 300 kDa and a degree of deacetylation of 80%, as well as 99% methanesulfonic acid, were purchased from Shanghai Mai Lin Biochemical Co. (Shanghai, China). Phosphite was supplied by the Aladdin Chemical Reagent Company (Shanghai, China). Hydrochloric acid and sodium hydroxide were provided by Liaoning Pulide Chemical Technology Co. (Shenyang, China) Lanthanum oxide (La_2_O_3_), dehydrated alcohol, acetone and a 37–40% formaldehyde solution were sourced from Chengdu Cologne Chemical Co., Ltd. (Chengdu, China). MD44 dialysis bags with a retention range of 8–14 kDa (average diameter 28 mm) were purchased from Shanghai Leibsey Company (Shanghai, China). All reagents used were of analytical grade.

### 2.2. Methods

#### 2.2.1. Synthesis of the aPCS

To improve the solubility and reactivity of chitosan, 5 g of chitosan was added to 25 g of sodium hydroxide dissolved in 75 mL of ultrapure water. The resulting mixture was stirred thoroughly at room temperature and stored in a refrigerator for 10 days. After thawing, the alkalized chitosan was collected by suction filtration, washed, and dried.

Weigh 0.5 g of the above alkalized chitosan dissolved in 150 mL of 5% methanesulfonic acid solution, stir thoroughly and transfer to a 500 mL round-bottomed flask, and then add 10 mL 40% phosphorous acid solution, stirring for 1 h to achieve full mixing. Increase the temperature to 75 °C, add an appropriate amount of 40% formaldehyde solution dropwise, and control the addition amount of phosphite and formaldehyde 1:1 (the molar ratio of amino groups in chitosan to phosphorous acid was approximately 1:17). The condensation and reflux reaction was then carried out for 7 h. After the reaction was completed, the solution was cooled to room temperature and dialyzed for 72 h (the MWCO of dialysis bag was 12,000 kDa). The dialysate was distilled under reduced pressure to obtain the product concentrate. A mixture of anhydrous ethanol and acetone was added to the concentrate, and a white flocculent precipitate was precipitated, which was allowed to stand overnight, filtered and washed, and then dried under vacuum at 55 °C to obtain the product, aPCS.

#### 2.2.2. Procedure of C–UF

In this process, La(III) was selected as a representative of rare earth ions. The ultrafiltration membrane device used in this study was the polyethersulfone (PES) hollow fiber membrane, with a molecular weight cutoff (MWCO) of 20 kDa. Firstly, the complexes were pretreated using diafiltration to remove small polymers. Then, complexant solutions of polymers with rare earths were prepared with different P/RE values (mass ratio of complexant to rare earth ions). After approximately 2 h of exposure, the prepared material was introduced into hollow fiber and flat plate membrane modules using a peristaltic pump operating at a feed rate of 30 L/h. Permeate samples were collected for 30 s after a 5 min interval and subsequently analyzed to measure the concentrations of rare earth ions and polymers. All experiments were performed at 25 °C.

### 2.3. Characterization

FTIR analysis (Nicolet iS50, Thermo Fisher Scientific, Waltham, MA, USA) was used to identify the functional groups present in the raw materials and the final products. The final products, dissolved in D_2_O, were analyzed using ^1^H NMR and ^31^P NMR spectroscopy (Bruker AVIII, 500 MHz, Zurich, Switzerland) to monitor and confirm the target compounds. The crystallinity of chitosan and aPCS was examined using XRD (Advance D8, Bruker AXS, Karlsruhe, Germany). The morphology of the products was observed by SEM on a JSM-7610F (JEOL, Tokyo, Japan) field emission scanning electron microscope. The concentration of La(III) in solution was determined using inductively coupled plasma optical emission spectrometry (ICP-OES, Avio 500, PerkinElmer, Singapore).

### 2.4. DFT Caculation

Structural optimization and single-point energy calculations of aPCS were performed using the B3LYP functional [21] with the 6-311G+** basis set [22]. The vibration frequencies are calculated after structural optimization. The DFT-D3 dispersion correction was applied to account for dispersion interactions, and the density-based implicit solvation model (SMD) was used to simulate the solvent environment. Then, the structures of aPCS–La (H_2_O)_6_^+^ complexes was optimized using the PBE0 functional [23] and the def2-SVP basis set [24]. All calculations were carried out using the Gaussian 16(C01) software package [25]. The electrostatic potential (ESP) of the aPCS molecule was then calculated using the Multiwfn 3.8 (dev) code [26] to analyze its properties and identify the optimal reaction site. The isosurface plots in this work were all realized by rendering cube files exported from the Multiwfn 3.8 (dev) code using visual molecular dynamics (VMD 1.9.3) software [27].

## 3. Results and Discussion

**Scheme 1:**
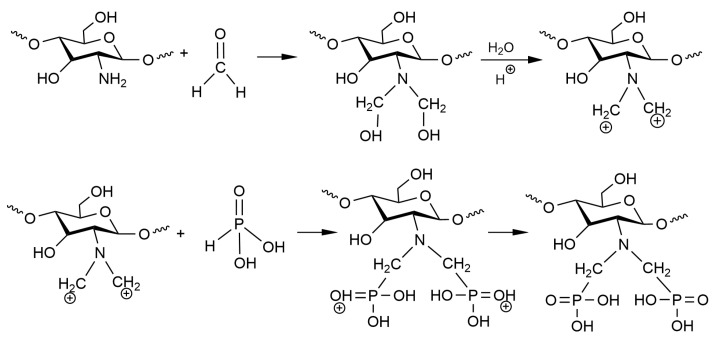
Synthesis mechanism for the aPCS.

Scheme 1 shows the synthesis mechanism of aPCS. The amine group of chitosan reacts with formaldehyde to form a positively charged iminium ion, which then undergoes electrophilic substitution with phosphorous acid. The resulting aPCS has a water solubility exceeding 2.9 mg mL^−1^, significantly higher than that of chitosan.

**Figure 1:**
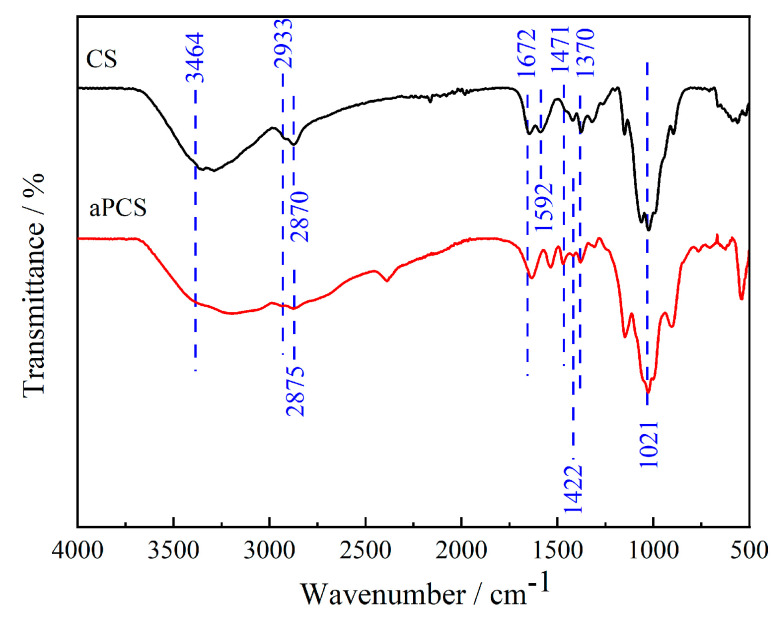
FTIR spectra of the aPCS and chitosan.

Figure 1 presents the FTIR spectra of the original chitosan and aPCS, CS presents a broad absorption band between 3464 and 3192 cm^−1^, which is attributed to the stretching vibrations of N-H and O-H bonds [28]. The asymmetric and symmetric stretching vibration absorption peaks observed at 2933 cm^−1^ and 2870 cm^−1^ are due to the presence of C-H bonds in -CH_2_- or -CH_3_- groups, respectively. The absorption peaks at 1672 cm^−1^ and 1592 cm^−1^ correspond to the stretching vibrations of C-O bonds and the in-plane bending vibrations of N-H groups. In the case of aPCS, the amine methylation reaction grafts -C-PO(OH)_2_ onto the C2-NH_2_ of CS, resulting in an intensified peak at 1471 cm^−1^. This peak is characteristic of the stretching vibrations of phosphonate groups (-C-PO(OH)_2_) and confirms the successful grafting of phosphonate moieties onto the molecular chain. Additionally, the broad absorption peak at 3464 cm^−1^ is retained but becomes even broader compared to CS, suggesting enhanced hydrogen bonding interactions. This broadening reflects the introduction of polar phosphonate groups, which increase the density and diversity of hydrogen bonding within the material, particularly in the non-crystalline regions. The hydroxyl absorption peak at 1370 cm^−1^ and the C-O bond absorption peak of the primary hydroxyl on C6 at 1021 cm^−1^ show minimal changes for aPCS, indicating that the modification primarily occurs at the C2-NH_2_ group and leaves the hydroxyl groups at C6 largely unaffected. A new absorption peak at 2875 cm^−1^ appears in aPCS, corresponding to the C-H stretching vibration of methylene groups (-CH_2_-), which confirms the involvement of formaldehyde in the amine methylation reaction. Furthermore, a strong peak at 1422 cm^−1^, attributed to the stretching vibration of C-P=O bonds, is observed, further supporting the successful introduction of phosphonate groups [29]. These spectral changes collectively indicate that phosphite and methylene groups have been grafted onto the molecular chain of chitosan, primarily at the amine functional groups.

**Figure 2:**
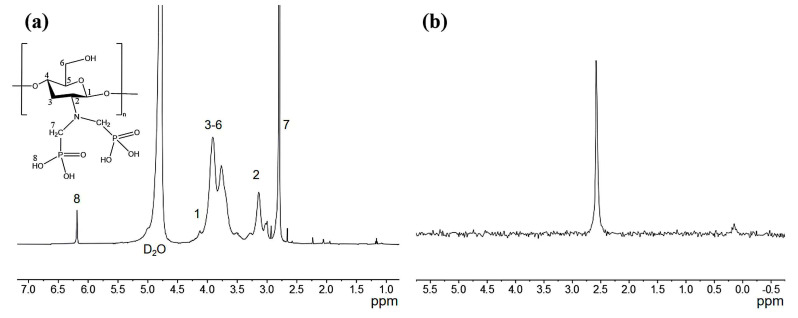
Schematic representation of the ^1^H NMR (**a**) and the ^31^P NMR spectrum of phosphonated chitosan (**b**).

The ^1^H-NMR and ^31^P-NMR of aPCS are presented in Figure 2a,b, respectively. As shown in Figure 2a, the signal at 4.5–4.8 ppm corresponds to H1, the peaks between 3.4 and 4.3 ppm are assigned to the H3–H6 protons, and the signal at 3.14 ppm is attributed to H2. These assignments are consistent with the ^1^H-NMR spectrum of chitosan reported in previous study [30]. For aPCS, the ^1^H-NMR spectrum displays a distinct peak at 2.65–2.94 ppm, corresponding to the methylene between phosphorus and nitrogen atoms, indicating that the methylene groups are grafted onto the amino position. Additionally, a new peak at 6.19 ppm is attributed to -C-PO(OH)2, confirming the attachment of the phosphate groups to the methylene groups. Furthermore, the ^31^P-NMR spectrum of aPCS, shown in Figure 2b, exhibits a prominent singlet at 2.56 ppm, providing direct evidence of phosphate groups incorporation.

**Figure 3:**
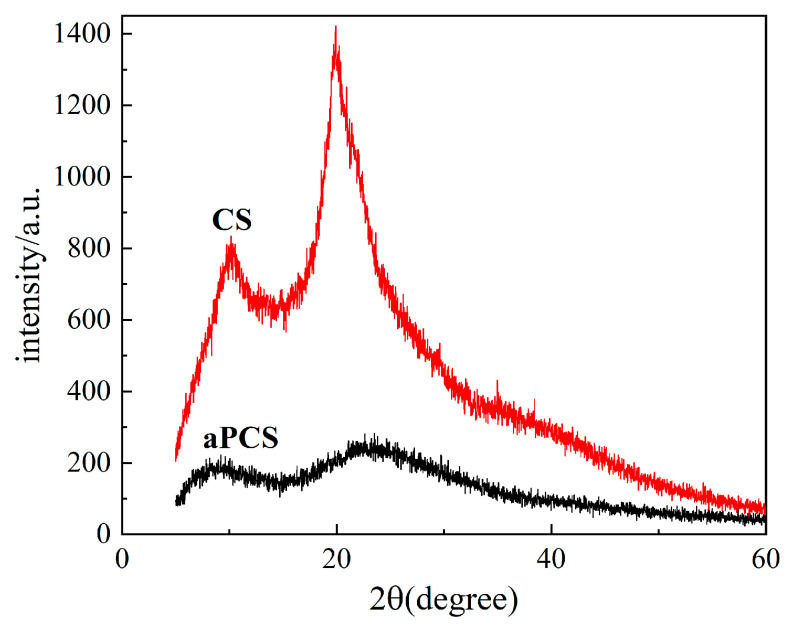
X-ray diffraction (XRD) patterns of the aPCS and CS.

The crystallinity of chitosan and aPCS is compared by XRD analysis, as shown in Figure 3. The pronounced diffraction peak at 20° for chitosan reflects its regular crystalline structure, driven by extensive hydrogen bonding. After modification, the diffraction intensity values found in aPCS decrease, indicating disruption of the crystalline structure of chitosan. This observation aligns with the FTIR results, where the newly introduced phosphonate groups weaken hydrogen bonding, reducing crystallinity and increasing molecular chain flexibility [31]. The increased molecular chain flexibility and reduced hydrogen bonding network improve the interaction of aPCS with water molecules, which directly contributes to its greater solubility in water compared to CS.

**Figure 4:**
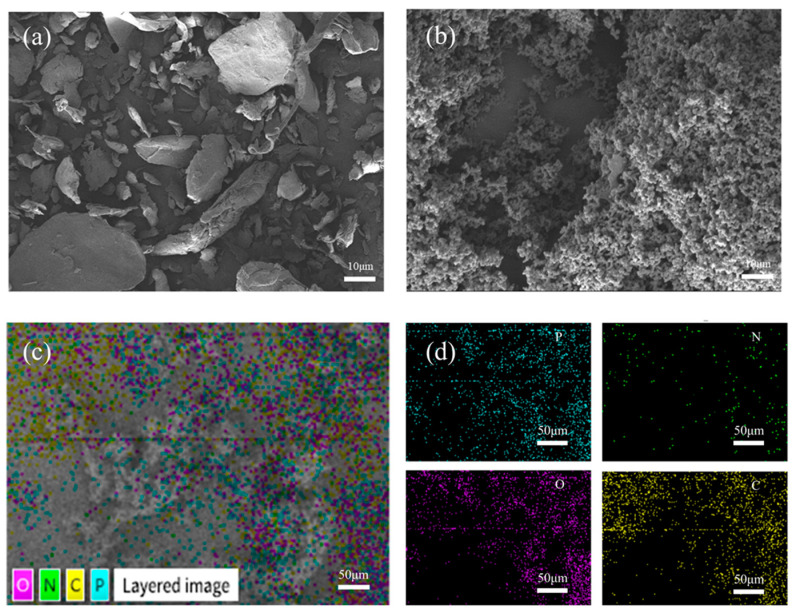
SEM images of CS (**a**) and aPCS (**b**), SEM-EDS mapping images of aPCS (**c**,**d**).

Figure 4a,b presents the SEM images of raw materials of chitosan and aPCS at the same magnification. The CS exhibits a regular shape with a smooth surface, while the surface of aPCS is rough and porous, indicating the structural changes caused by the grafting of phosphonate groups. This disrupted crystalline structure correlates with the enhanced water solubility of aPCS. Concurrently, the element distribution of aPCS is analyzed by SEM–EDS mapping (Figure 4c,d). The product contains elements P, N, O, and C, with a higher quantity of P compared to N, the target material has been successfully synthesized.

**Figure 5:**
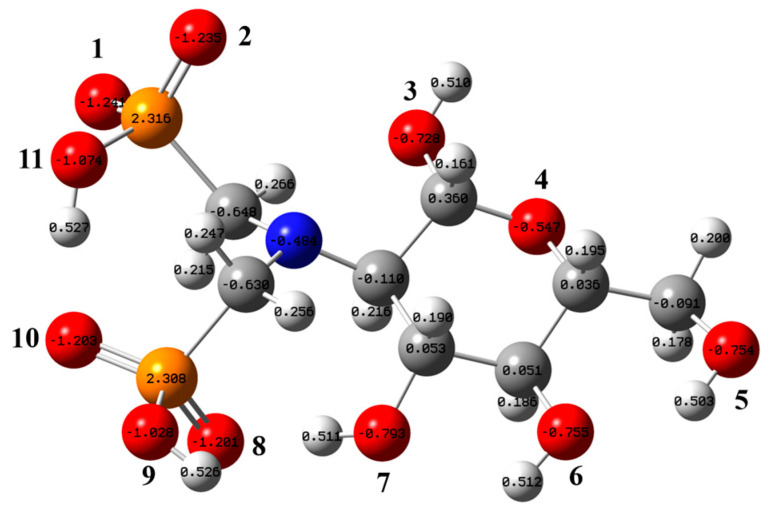
Optimized stable structure of aPCS and the NPA atomic charge distribution (white, gray, blue, red, and orange spheres represent H, C, N, O, and P atoms, respectively; only the oxygen atoms are labeled).

Figure 5 presents the most stable structure of the aPCS molecule, with vibrational frequency analysis confirming the absence of imaginary frequencies, indicating that the optimized structure is the true global minimum on its potential energy surface. The figure also shows the natural population analysis (NPA) charge distribution of aPCS. Atomic charges provide a simple and intuitive way to describe charge distribution in a system. Atoms with stronger negative charges are more likely to attract positively charged nucleophiles, thereby facilitating chemical reactions [32]. In the aPCS molecule, the oxygen atoms O(1), O(2), O(8), and O(10) of the phosphonate groups exhibit the strongest affinity for rare earth cations. Electron delocalization within the phosphonate group leads to an equal distribution of negative charge across O(1) and O(2). However, the hydrogen bond between O(10) and H-O(11) weakens the electronegativity of O(8) and O(10) compared to O(1) and O(2), which are part of the same phosphonate group.

**Figure 6:**
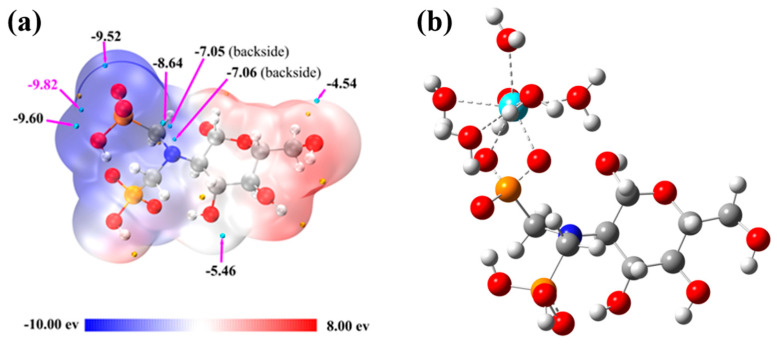
Distribution of ESP on the surface of aPCS molecules (**a**) and stable structure of aPCS–La (H_2_O)_6_^+^ after optimization (cyan sphere represents La atom) (**b**).

Given that the interaction between trivalent rare earth ions and organic ligands is predominantly ionic, ESP analysis is crucial for identifying the ligand’s reactive sites and evaluating coordination strength. By analyzing the ESP on the ligand surface, regions likely to attract rare earth ions can be predicted [3]. Figure 6a shows the ESP distribution on the van der Waals surface of the aPCS molecule, with negative potential (blue) indicating areas susceptible to electrophilic attack, and positive potential (red) indicating nucleophilic attack sites. Cyan and yellow spheres represent the minima and maxima of the electrostatic potential, respectively, with the global minimum highlighted in magenta. The van der Waals surface of the phosphonate groups is predominantly deep blue or white, with no significant red regions, and the global minimum of the electrostatic potential (−9.82 eV) is located in this region. This suggests that this portion of the surface is electron-rich due to deprotonation, forming an anionic system that has a stronger attraction to rare earth cations. In our previous work, it was demonstrated that PCS primarily attracts rare earth ions through -NH and -P=O groups, subsequently forming coordination bonds [33].

Figure 6b shows the optimized structure of the complex after placing La(III) at the global minimum binding site of aPCS, visually illustrating the complexation mechanism between aPCS and La(III). The results are consistent with the ESP analysis, which suggests that the phosphonate groups in aPCS, upon deprotonation to form an anionic system, not only attract rare earth ions via long-range electrostatic interactions but also tend to coordinate in a bidentate manner with rare earth cations [34], thereby forming more stable complexes than previously synthesized PCS.

Building on the DFT analysis, which revealed favorable structural and electronic properties of aPCS for rare earth ion complexation, we experimentally investigated its ability to complex La(III).

**Figure 7:**
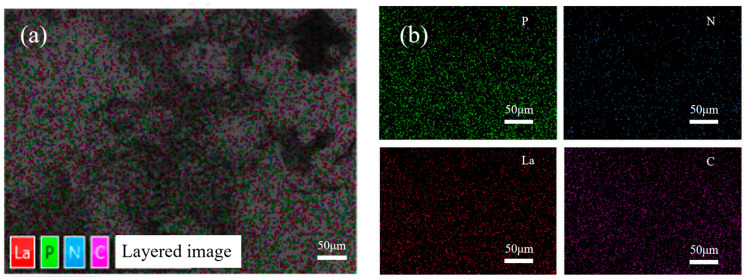
SEM-EDS mapping image showing the element distribution of aPCS-La complexes (**a**) and individual elemental mapping images corresponding to P, N, La, and C (**b**).

Figure 7 shows the elemental distribution of the aPCS–La complex as analyzed by SEM–EDS mapping. The P element remains abundant, consistent with the structure of aPCS. Notably, La exhibits a spatial distribution that closely overlaps with that of P. This spatial overlap indicates that La(III) are coordinated to the phosphonate groups of aPCS, providing preliminary evidence for the formation of aPCS-La complexes.

**Figure 8:**
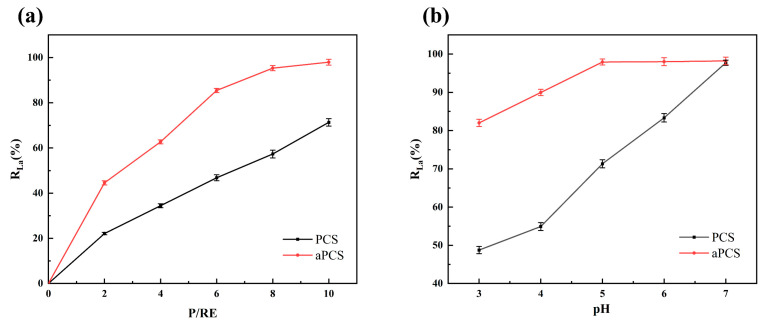
Comparison of complexing properties between aPCS and PCS at pH 5 (**a**), P/RE 10 (**b**).

Ultrafiltration membranes were used to retain the aPCS-La complexes. The rejection rate (R), which represents the effect of complexation, was calculated as the ratio between the concentration of La(III) in the retentate and that in the original solution. As shown in Figure 8a, at pH 5 and P/RE 6, aPCS achieved a rejection rate exceeding 85% for La(III), compared to only 45% for PCS. When the P/RE ratio was increased to 10, the number of complexation sites provided by aPCS was sufficient to coordinate La(III) in solution. At this ratio, aPCS achieved a significantly higher rejection rate of 97%, compared to 70% for PCS. Furthermore, as shown in Figure 8b, the rejection rates of aPCS and PCS were evaluated at P/RE 10 across different pH, with aPCS consistently outperforming PCS. Additionally, the results of the ultrafiltration experiments provide strong evidence that La(III) ions are effectively complexed with both aPCS and PCS. This is demonstrated by the significantly lower concentration of rare earth ions in the permeate compared to the feed solution, indicating that the majority of the ions were retained as stable complexes. The superior performance of aPCS under acidic conditions can be attributed to the dissociation of hydrogen atoms from its phosphonate groups, forming an anionic system that enhances electrostatic interactions with La(III). The structural features of aPCS effectively promote the formation of stable complexes with La(III) at the acidic pH range of 3 to 7. These results underscore the potential of aPCS as an efficient ligand for rare earth recovery in industrial applications, particularly under acidic conditions.

## 4. Conclusions

In summary, aPCS has been successfully synthesized as a highly effective complexing agent for enhancing its complexation performance of rare earth ions in acidic aqueous solutions. The modification of chitosan with phosphonate groups significantly improves its water solubility and introduced functional sites capable of coordinating with rare earth elements. DFT analysis revealed that aPCS possesses favorable for bidentate coordination with La(III), indicating a strong binding affinity and structural stability of the resulting complexes. Experimental results confirmed that at pH 5, aPCS achieves a remarkable rejection rate of 97% for La(III), demonstrating its superior complexation performance in comparison to PCS. Moreover, aPCS maintains higher rejection rates than PCS within the acidic pH range of 3 to 7, emphasizing its adaptability to varying conditions. The preparation of aPCS is straightforward, making it a promising candidate for the selective separation and purification of rare earth ions in acidic aqueous solutions.

## Data Availability

Data are contained within the article.
